# Supplementary figures and images for: Plasticity of the proteasome-targeting signal Fat10 enhances substrate degradation
Source: eLife. 2024 Jul 10;13:e91122. doi: 10.7554/eLife.91122 (PMC11299979; doi:10.7554/eLife.91122)

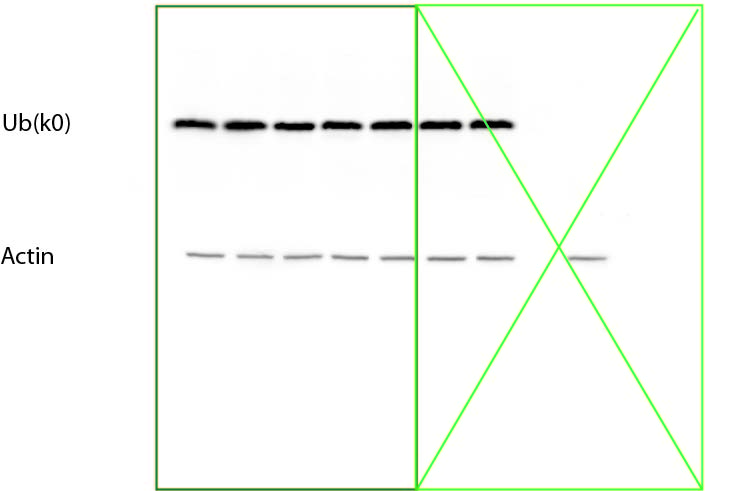

Supplement: Figure 1—source data 1. [file elife-91122-fig1-data1.zip › Figure1-Source Data 1/Figure1D_sourceData1.jpg]

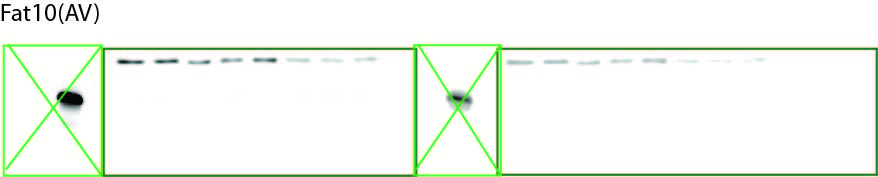

Supplement: Figure 1—source data 1. [file elife-91122-fig1-data1.zip › Figure1-Source Data 1/Figure1D_sourceData2.jpg]

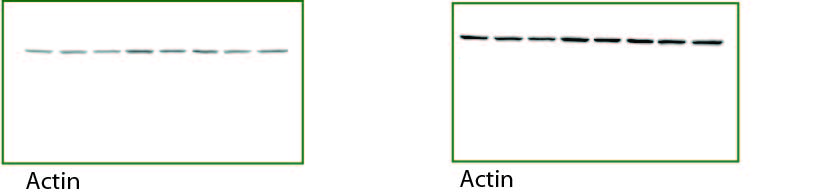

Supplement: Figure 1—source data 1. [file elife-91122-fig1-data1.zip › Figure1-Source Data 1/Figure1D_sourceData3.jpg]

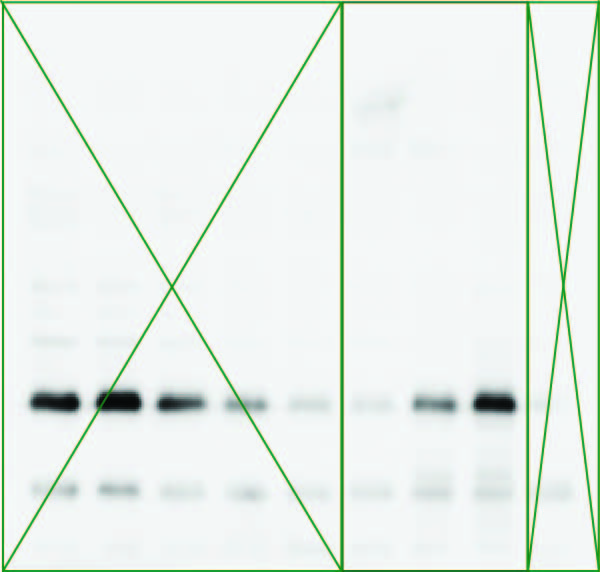

Supplement: Figure 1—source data 1. [file elife-91122-fig1-data1.zip › Figure1-Source Data 1/Figure1F_sourceData1.jpg]

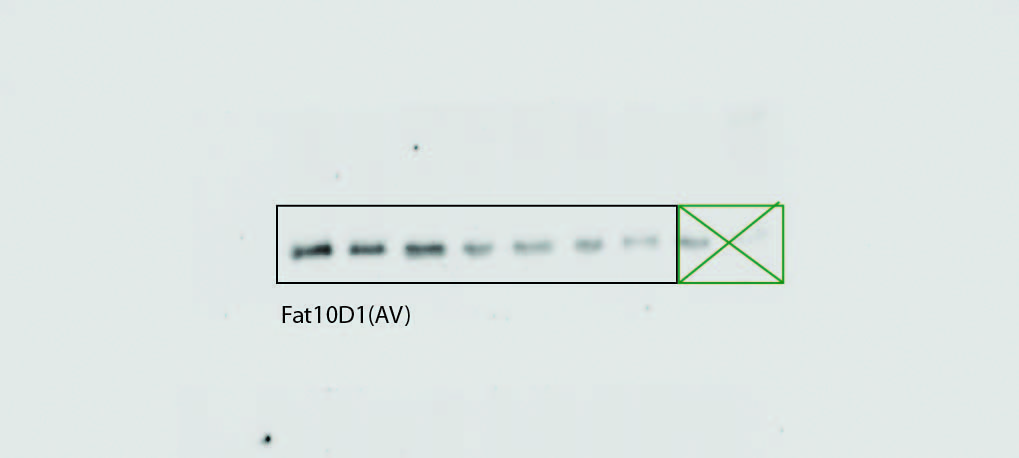

Supplement: Figure 1—source data 1. [file elife-91122-fig1-data1.zip › Figure1-Source Data 1/Figure1G_sourceData1.jpg]

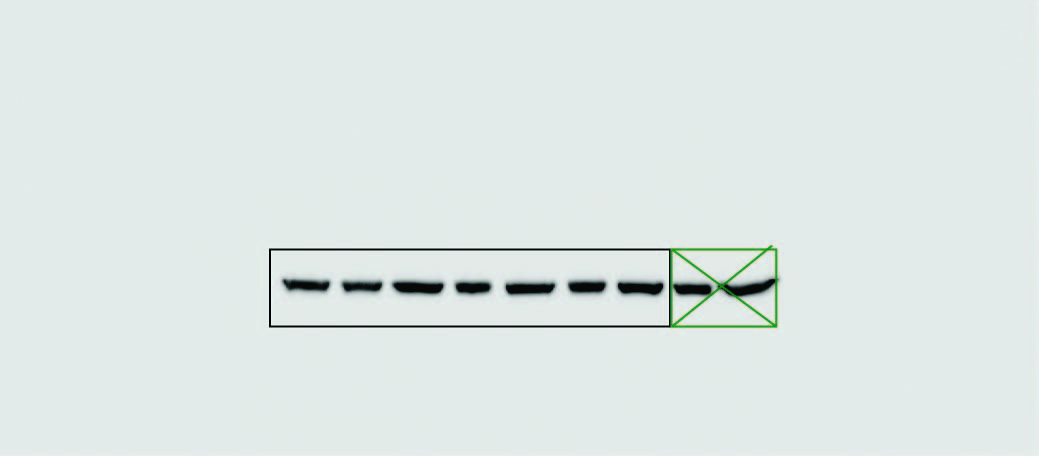

Supplement: Figure 1—source data 1. [file elife-91122-fig1-data1.zip › Figure1-Source Data 1/Figure1G_sourceData2.jpg]

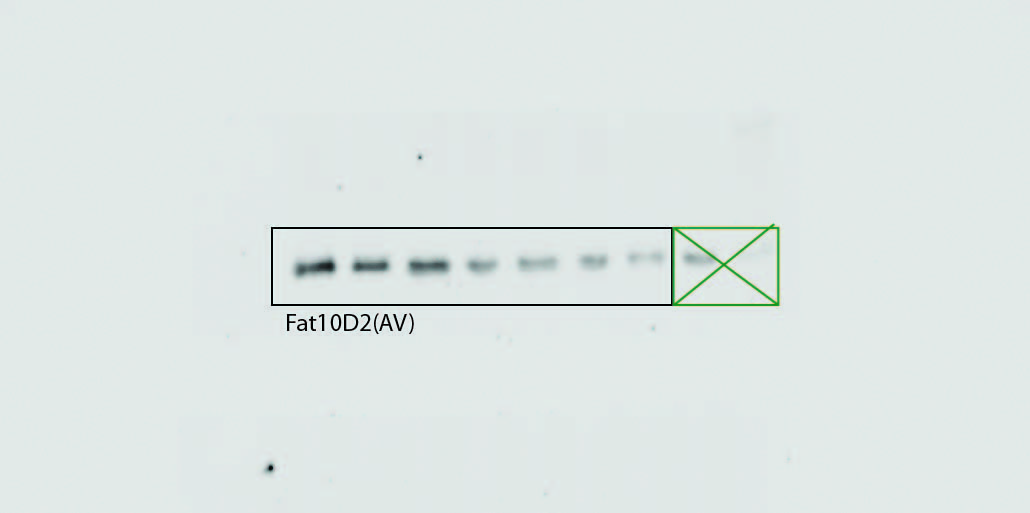

Supplement: Figure 1—source data 1. [file elife-91122-fig1-data1.zip › Figure1-Source Data 1/Figure1H_sourceData1.jpg]

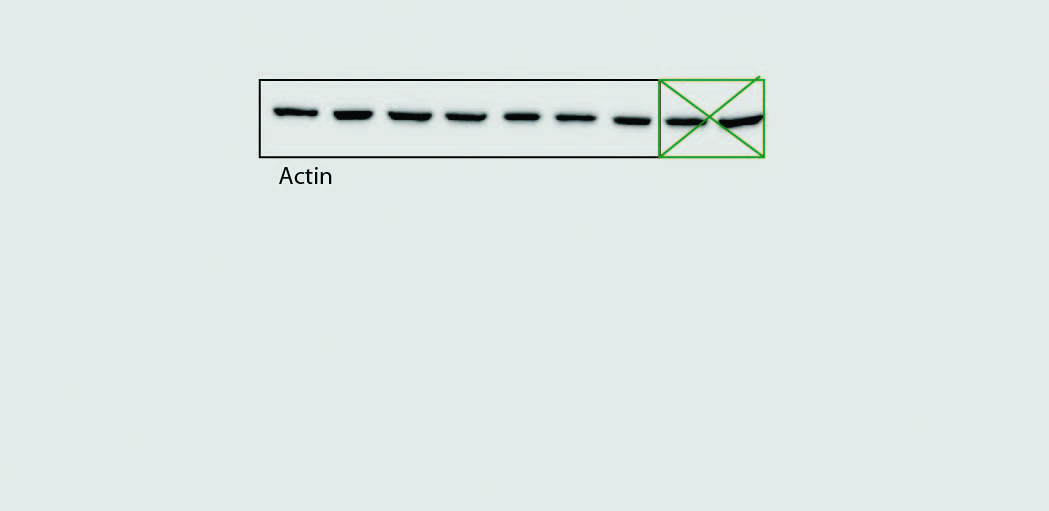

Supplement: Figure 1—source data 1. [file elife-91122-fig1-data1.zip › Figure1-Source Data 1/Figure1H_sourceData2.jpg]

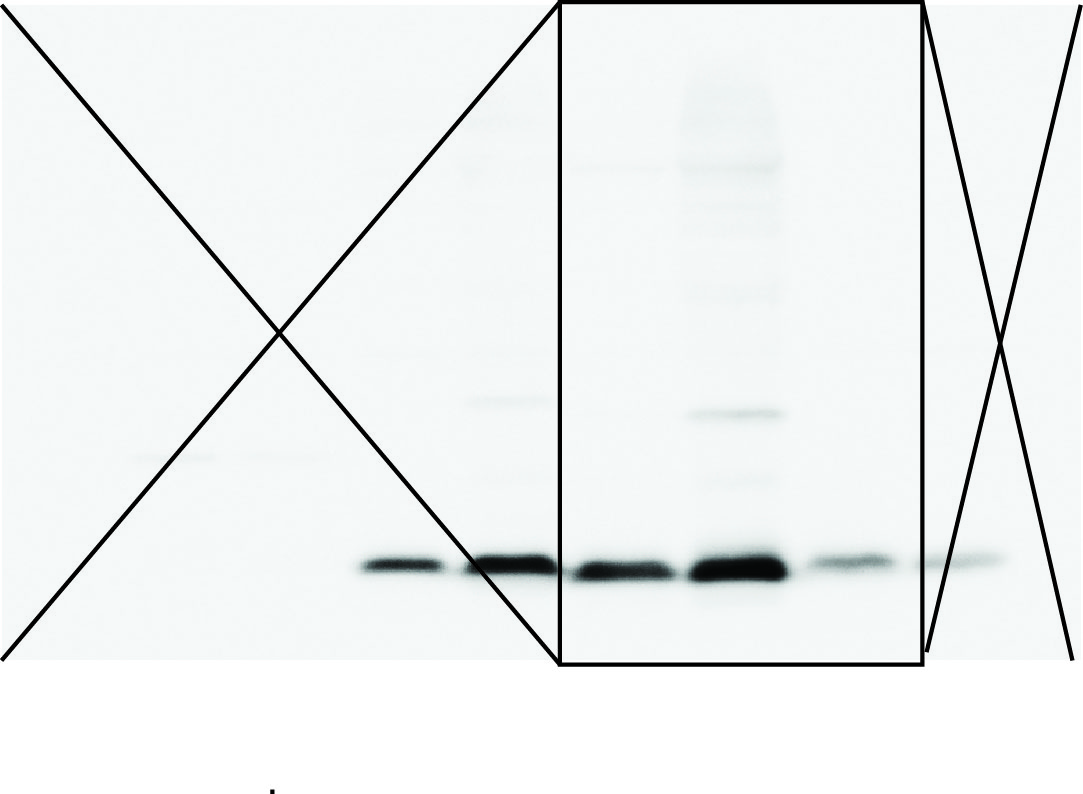

Supplement: Figure 1—figure supplement 1—source data 1. [file elife-91122-fig1-figsupp1-data1.zip › Figure1-Source Data 2/SuppFig1F-SourceData1.jpg]

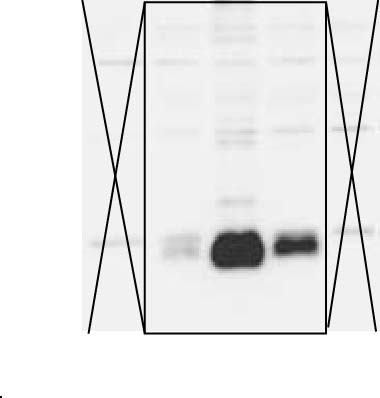

Supplement: Figure 1—figure supplement 1—source data 1. [file elife-91122-fig1-figsupp1-data1.zip › Figure1-Source Data 2/SuppFig1G-SourceData1.jpg]

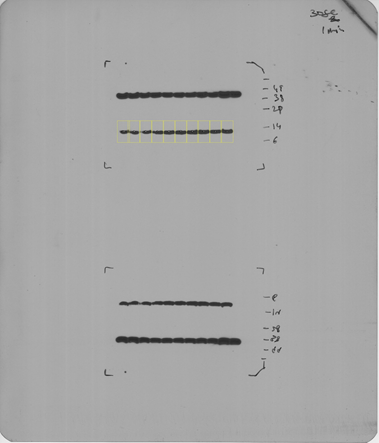

Supplement: Figure 1—figure supplement 1—source data 2. [file elife-91122-fig1-figsupp1-data2.zip › Figure 1-Source Data 3/Figure 1-Source Data 3.tif]

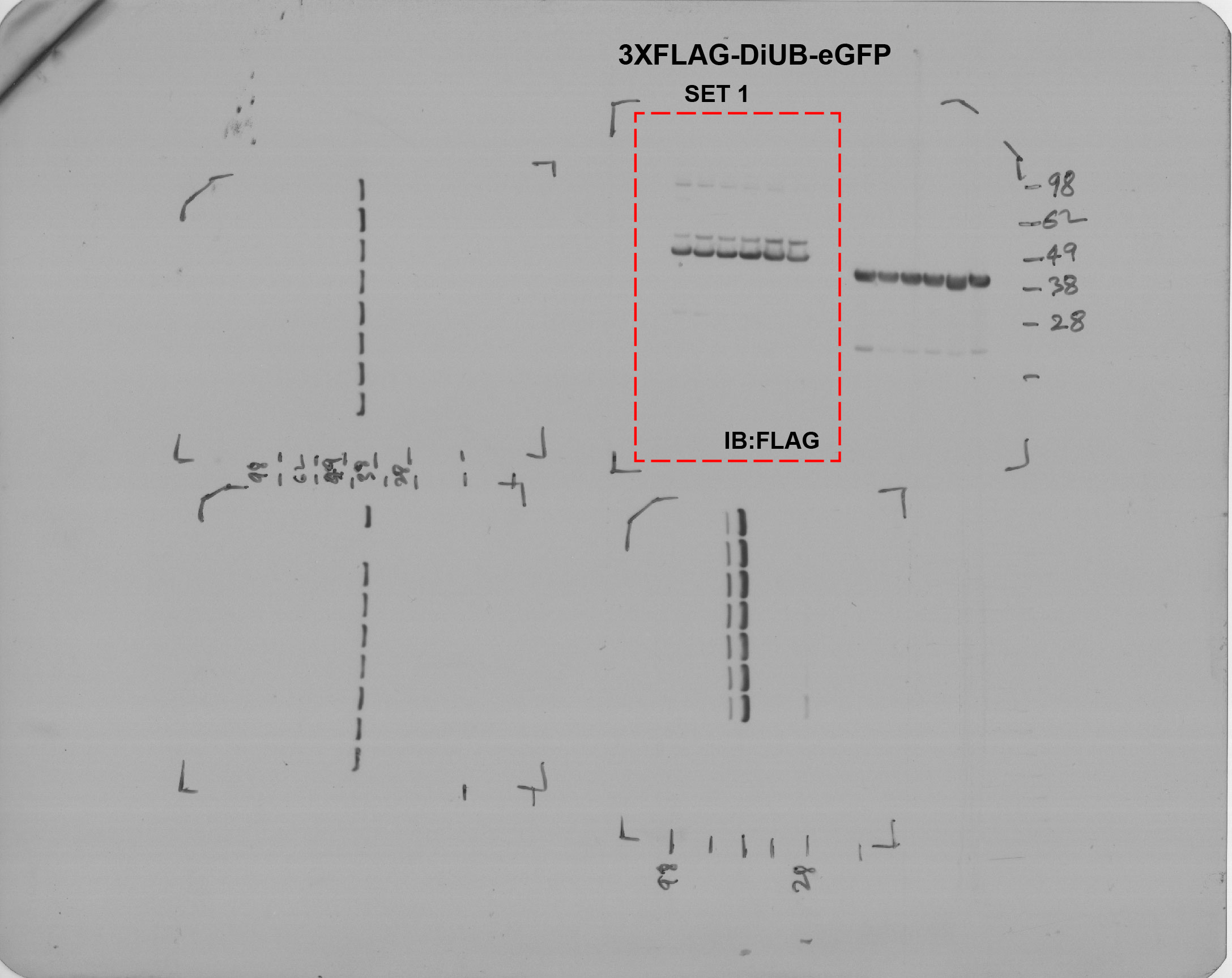

Supplement: Figure 5—source data 1. [file elife-91122-fig5-data1.zip › Figure 5-Source Data 1/Fig5_supplemental1A_sourcedata.jpg]

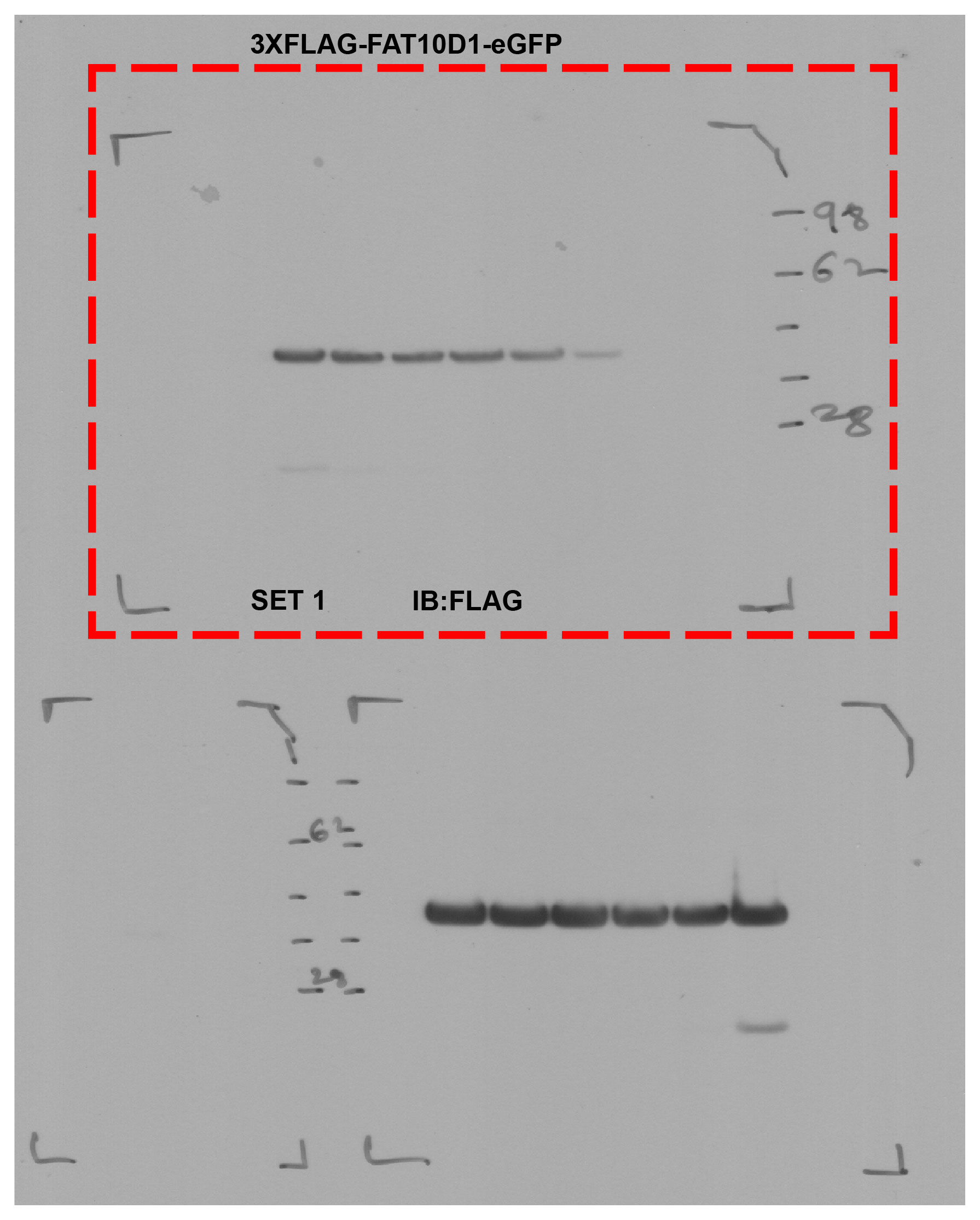

Supplement: Figure 5—source data 1. [file elife-91122-fig5-data1.zip › Figure 5-Source Data 1/Figure5_supplemental1B1_sourcedata.jpg]

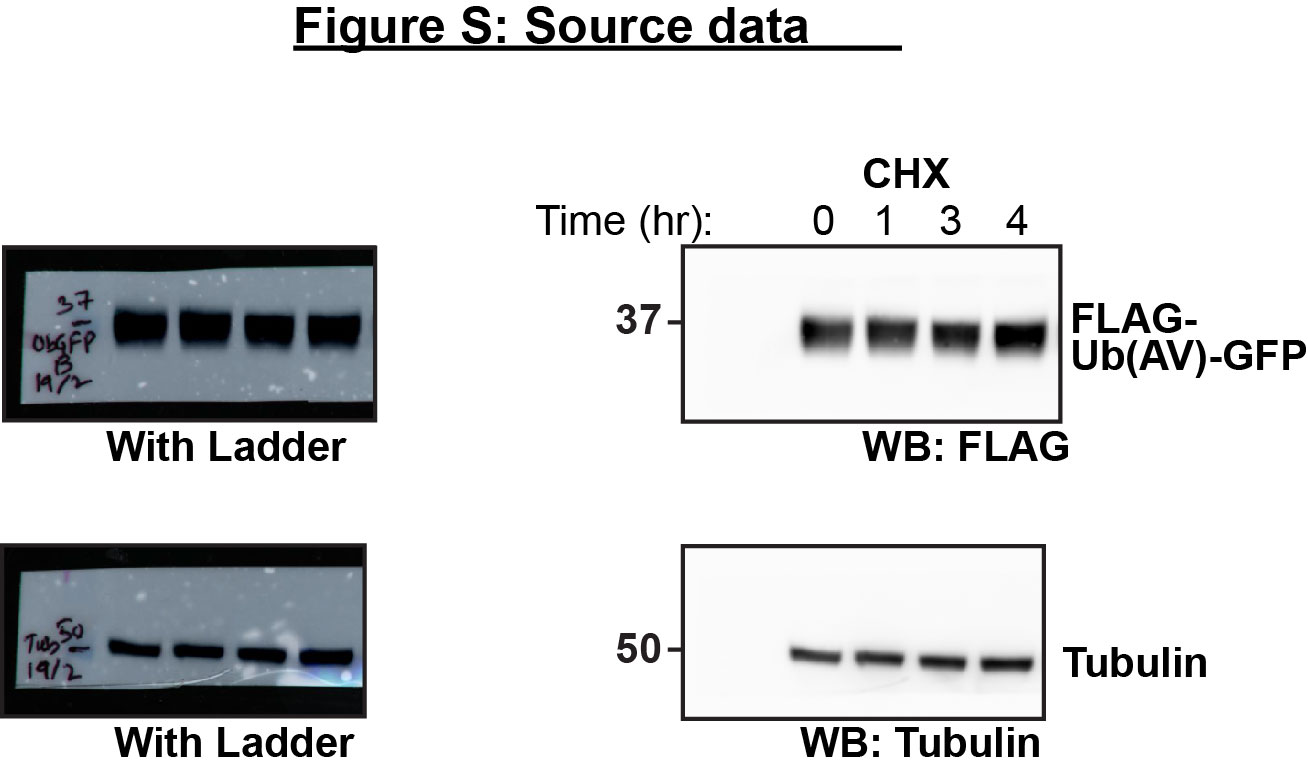

Supplement: Figure 5—source data 1. [file elife-91122-fig5-data1.zip › Figure 5-Source Data 1/Figure5_Supplemental1B_SourceData.jpg]

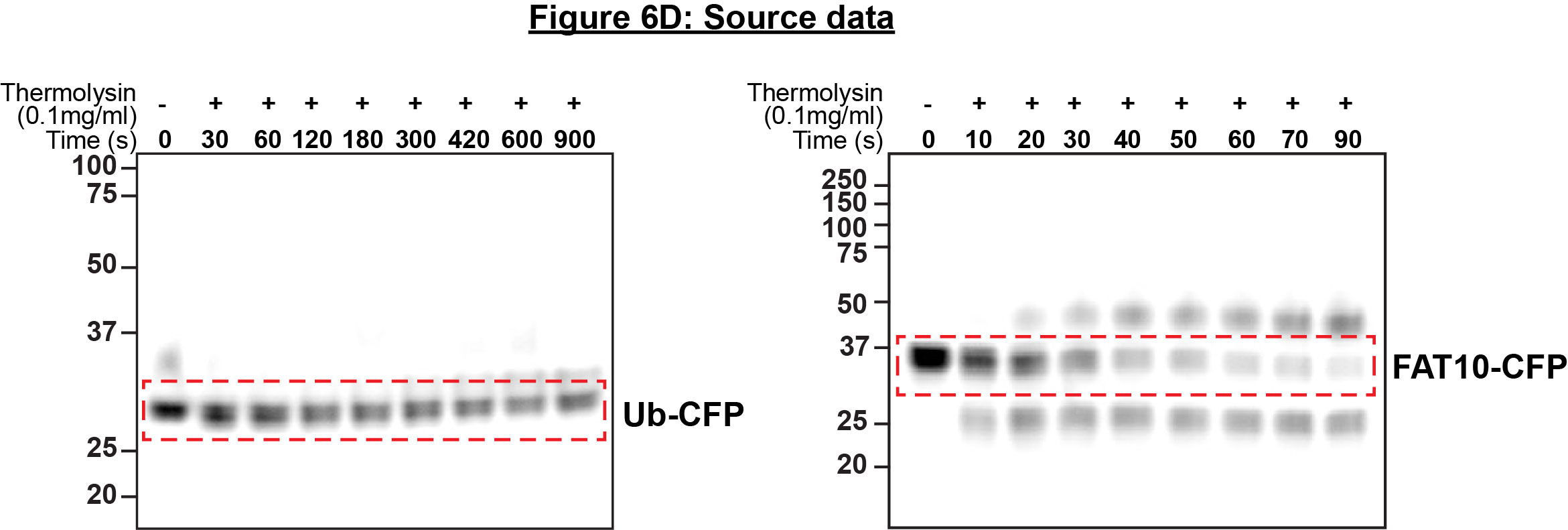

Supplement: Figure 6—source data 1. [file elife-91122-fig6-data1.zip › Figure 6-Source Data 1/Figure 6D_Source data.jpg]

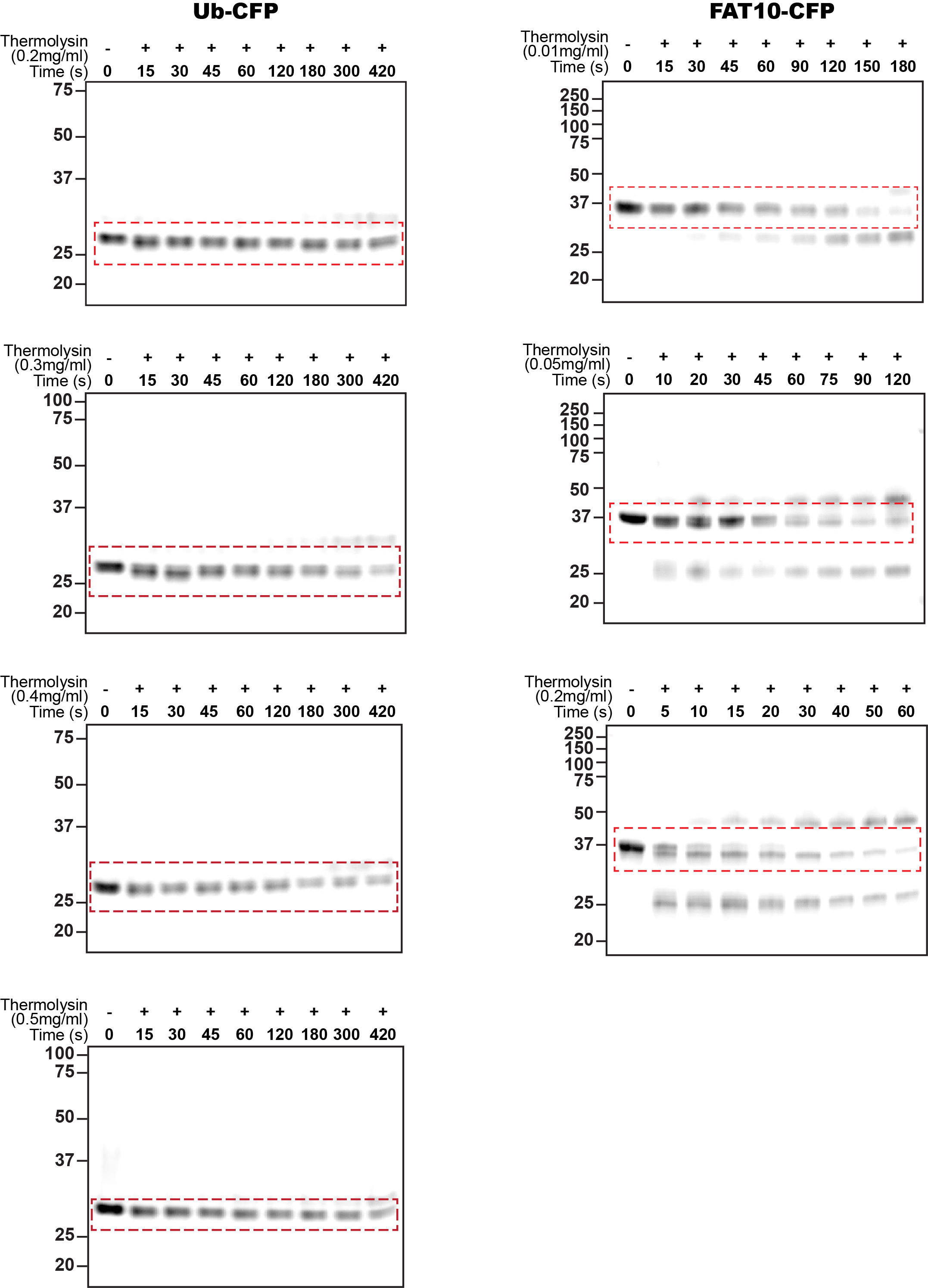

Supplement: Figure 6—source data 1. [file elife-91122-fig6-data1.zip › Figure 6-Source Data 1/Figure6_Supplemental1_Source.jpg]

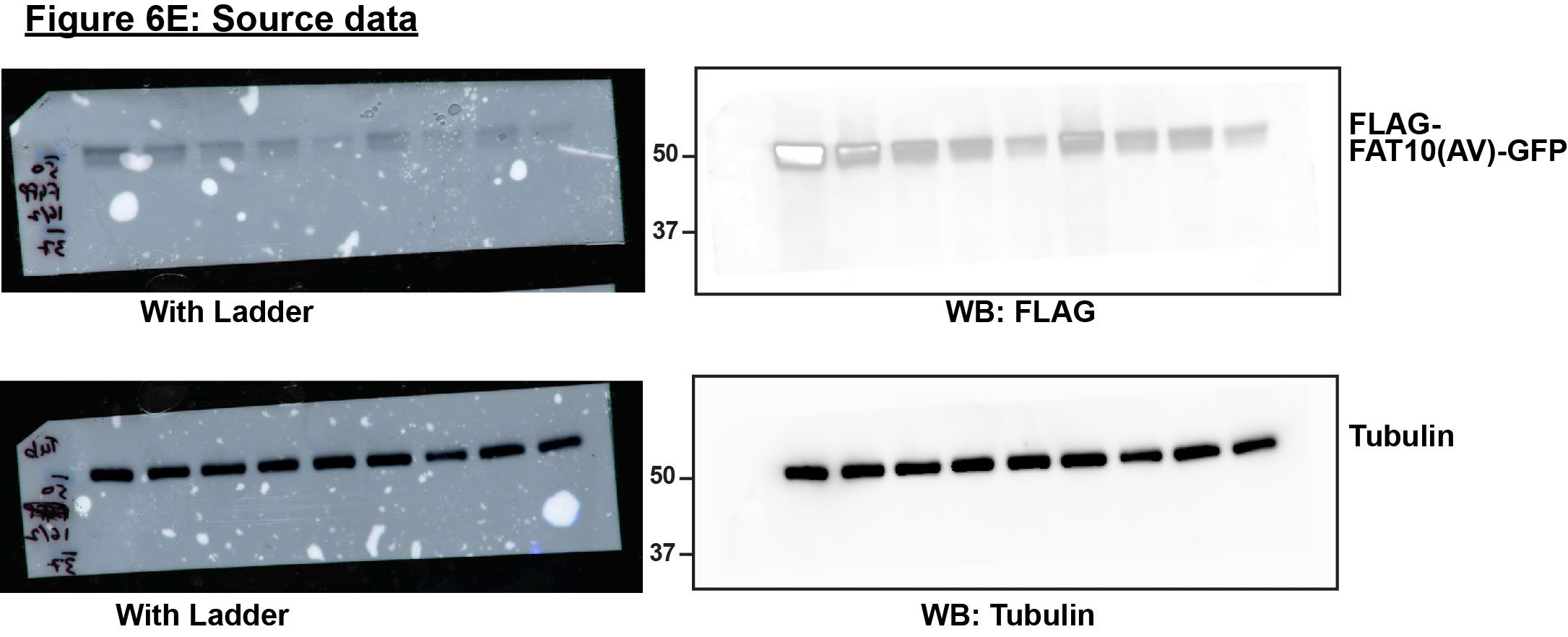

Supplement: Figure 6—source data 2. [file elife-91122-fig6-data2.zip › Figure 6-Source Data 2/Figure 6E_Source data.jpg]
